# Suitability Assessment of PLA Bottles for High-Pressure Processing of Apple Juice

**DOI:** 10.3390/foods10020295

**Published:** 2021-02-02

**Authors:** Arianna Cubeddu, Patrizia Fava, Andrea Pulvirenti, Hossein Haghighi, Fabio Licciardello

**Affiliations:** 1 Department of Life Sciences, University of Modena and Reggio Emilia, 42122 Reggio Emilia, Italy; arianna.cub@hotmail.com (A.C.); patrizia.fava@unimore.it (P.F.); andrea.pulvirenti@unimore.it (A.P.); hossein.haghighi@unimore.it (H.H.); 2 Interdepartmental Research Centre BIOGEST-SITEIA, University of Modena and Reggio Emilia, 42124 Reggio Emilia, Italy

**Keywords:** apple juice, color, compostable packaging, headspace gas composition, high-pressure processing, microbiological stability, non-destructive gas measurement, polyethylene terephthalate, polylactic acid, shelf life

## Abstract

The aim of the present study is to assess the use of polylactic acid (PLA) bottles as an alternative to polyethylene terephthalate (PET) ones for high-pressure processing (HPP) of apple juice. The treatment of PLA bottles at 600 MPa for 3 min did not cause alterations in the packaging shape and content, confirming the suitability of PLA bottles to withstand HPP conditions as well as PET bottles. Quantification of total mesophilic bacterial and fungal load suggested HPP treatment can be effectively applied as an alternative to pasteurization for apple juice packed in PLA bottles since it guarantees microbial stability during at least 28 days of refrigerated storage. The headspace gas level did not change significantly during 28 days of refrigerated storage, irrespective of the bottle material. Color parameters (L*, a*, and b*) of the HPP-treated juice were similar to those of the fresh juice. Irrespective of the packaging type, the total color variation significantly changed during storage, showing an exponential increase in the first 14 days, followed by a steady state until the end of observations. Overall, PLA bottles proved to offer comparable performances to PET both in terms of mechanical resistance and quality maintenance.

## 1. Introduction

High-pressure processing (HPP), also known as the ultra-high pressure (UHP) or high hydrostatic pressure (HHP) process, is an innovative, non-thermal technology that has been recently adopted as an alternative to traditional thermal methods for many products as well as fruit juice [1,2,3]. In this technology, the product is subjected to a very high level of hydrostatic pressure (up to 1000 MPa) for a short period (ranging from a few seconds to a few minutes) to inactivate microorganisms, denaturing endogenous enzymes, preserving the sensory characteristics, flavors, and nutritional components, such as vitamins [4,5]. The principle of this technology is based on the uniform transmission of the pressure to the entire mass of the product thanks to the water contained in the hyperbaric chamber and in the product itself. Because of the uniformity of the pressure distribution, this method does not cause damage or distortion to the products, as long as they do not have a porous or hollow structure [4]. It allows the inactivation of microorganisms, as well as preserving vitamins, color, and flavors [6]. It was reported that pressures ranging from 300 to 600 MPa for 1 to 15 min inactivate the vegetative microflora (bacteria, viruses, yeasts, and molds) present in food [6]. However, this technique without heat treatment is not effective in the inactivation of spores and enzymes [7,8]. Endogenous enzymes often have a higher resistance to high-pressure conditions than the vegetative forms of spoilage bacteria [9].

HPP products must be packaged in flexible or semi-flexible packaging, which can withstand very high pressures and can allow the transfer of pressure from the pressure transmission fluid to the food or beverages contained in the package [10,11]. To date, HPP processes are applied on foods packed in plastic materials, such as polyethylene (PE), polypropylene (PP), and polyethylene terephthalate (PET), that can withstand the extreme conditions required by the treatment, while maintaining the key functional parameters (mechanical resistance, integrity, barrier, etc.).

Similar to other perishable fruits, apples are either consumed fresh or processed into various value-added products, such as juice, pulp, syrup, sauce, jam, jelly, pickles, and powders [12]. Among these products, apple juice production is one of the most economical and easy ways for value addition [13]. The high content of antioxidants (e.g., polyphenols, especially flavonoids), vitamins (e.g., ascorbic acid), dietary fibers (e.g., pectin), and the low levels of sodium, fat, and calories [14] justify its consideration as part of a healthy diet since the end of the 19th century when the saying “an apple a day keeps the doctor away” started to become popular [15].

The market of fruit juices is dominated by ultra-high temperature processing (UHT) products. In the UHT technique, fruit juices are heat-treated at high temperatures (above 100 °C) for a few seconds and cooled rapidly. Applying this technique, apple juices can be stored for a long time at room temperature. This technique is quite efficient in preventing microbial spoilage and enzyme activity, leading to maintaining safety and extending the shelf life [16]. However, high temperatures may also cause the loss of nutrition and volatile compounds, sensory properties, the formation of unwanted substances, damage to bioactive constituents, color and aromatic changes that may affect the overall quality of the final product [17,18].

Recently, a growing number of consumers demand not only the quality of the food but also environmentally friendly and sustainable food packaging materials [19]. Amienyo et al. [20] reported that carbonated soft drinks packed in PET bottles (even if these materials are perfectly recyclable) are characterized by a high packaging relative environmental impact (PREI) ranging from 49 to 59 (expressed as the percentage of global warming potential, GWP%). PREI is defined as the ratio between the impact (expressed as carbon footprint) relating to packaging and that attributable to the food production stages [19]. For these types of products, the improvement of the environmental performance of the product can be achieved through the reduction of the packaging impact rather than interventions to improve the efficiency of the beverage production processes [21]. The use of biobased food packaging materials could meet the objectives required by the U.S Food and Drug Administration (FDA) and the European Union in terms of sustainability [22,23].

Polylactic acid (PLA) is an aliphatic bio polyester that can be synthesized either by carbohydrate fermentation (e.g., corn starch or sugarcane) or by chemical synthesis of the lactic acid monomer [24]. PLA is categorized as GRAS (generally recognized as safe) and approved by the FDA and the European Community’s Scientific Committee on Food for fabricating materials in direct contact with food [25,26]. Various studies investigated the suitability of PLA biopolymer as food packaging material in the manufacturing of HPP products [11,27,28], suggesting that HP pasteurization does not significantly affect the structural and functional properties of the packaging material and that PLA does not interact at a significant extent with the food, resulting in low migration of additives and low scalping of aroma compounds at HP pasteurization conditions. To the best of our knowledge, the literature on the evaluation of suitability of PLA bottles for the packaging and HPP treatment of juices is lacking. Therefore, the present study aimed to assess the possible use of PLA bottles as an alternative to PET ones for the packaging and HPP treatment of apple juice. For this purpose, PET and PLA bottles with identical geometric characteristics were compared and the ability of PLA bottles to withstand the mechanical stress related to the HPP was analyzed. Besides, the qualitative characteristics of cold stabilized apple juice, including color coordinates (L*, a*, and b*), total color variation, yellowness index, headspace gas composition, and quantification of total mesophilic bacterial and fungal load, were assessed during 28 days of refrigerated storage.

## 2. Materials and Methods

### 2.1. Materials

Apple juice was kindly supplied by Tuscan Juicery Srl (Cortona, Italy) in 5 L bag in a box at refrigerated conditions (4 °C) and was immediately bottled as received. PET and PLA bottles (500 mL) with polyethylene screw caps (38 mm diameter) were kindly supplied by PetPack s.r.l. (Predosa, Italy). PET and PLA bottles were blow-molded using the same molds to have identical geometrical features. Blowing of pre-heated parisons was performed with compressed air at 25–40 bar in molds, which were cooled with water at 7–8 °C. Bottles had an average weight of 24.5 ± 0.5 g and an average thickness of 1.00 ± 0.30 mm.

### 2.2. Samples Preparation and High-Pressure Processing

PET and PLA bottles (500 mL) were filled with apple juice (approx. 490 mL) and flushed for 30 s with nitrogen and capped in a nitrogen saturated chamber to allow the reduction of the amount of oxygen and its replacement with nitrogen. High hydrostatic pressure (HPP) treatment of samples was carried out with QFP 350L-600 equipment (Avure Technologies Inc., Kent, WA, USA) supplied with a 350 L hyperbaric chamber at Hpp Italia s.r.l (Traversetolo, Italy). Bottles were manually loaded into the baskets and sent to the hyperbaric chamber for pressurization at 600 MPa for 3 min at 6 °C with a thermally insulated steel holder to minimize heat losses followed by a rapid depressurization. The indicated HPP treatment time does not include come-up and come-down times. Pressure monitoring was performed via a PanelView ™ Plus 6 1250 series graphic terminal (Allen-Bradley, Milan, Italy). The pressurized and control (untreated) samples were transferred to the laboratory, where they were kept in refrigerated conditions (6 ± 1 °C) through the duration of the tests and sampled at regular intervals up to 28 days (0, 7, 14, 21, and 28 days).

### 2.3. Microbiological Analyses

Microbiological analysis was determined 12 h after HPP treatment and after 7, 14, 21, and 28 days of refrigerated storage at 6 °C. Appropriate dilutions were made with sterile saline solution (0.9% NaCl) and one mL of each dilution was plated onto the culture media. Total mesophilic aerobic bacteria were determined using Plate Count Agar (PCA, Biolife, Milan, Italy) after incubation at 30 °C for 48 h. Yeasts and molds were counted in Sabouraud Dextrose Agar (SDA, VWR Chemicals, Milan, Italy) after incubation at 20 °C for 48 h. The results are expressed as the colony-forming units per mL of apple juice (CFU/mL).

### 2.4. Headspace Gas Composition Analysis

#### 2.4.1. Conventional Approach by Gas-Chromatography

A GC 320 gas chromatograph (GL Sciences, Tokyo, Japan) equipped with a thermal conductivity detector and equipped with a CTR column (Alltech^®^ IC, Sedriano, Milan, Italy) for the separation of O_2_, N_2_, CH_4_, CO, CO_2_ was used for detection. A total of 50 μL gas was withdrawn from the bottle headspace with a 100 μL gas-tight syringe (Hamilton, CA, USA) and injected into the column at 55 °C. The carrier gas was helium with a flow rate of 1 mL/min. The identification of the chromatographic peaks was performed by comparison with the retention times of the peaks related to known standards. The quantitative determinations of the gases were based on the comparison between the areas of the peaks obtained and those of the peaks produced by known volumes of pure gas. The analysis of the gas composition was carried out at regular intervals up to 28 days (0, 7, 14, 21, and 28 days) in duplicate on PET and PLA bottles before opening for further analysis. The results were reported as O_2_% and CO_2_%.

#### 2.4.2. Non-Destructive Determination by Laser Spectroscopy

Empty and water-filled bottles were monitored for the headspace O_2_ and CO_2_ concentrations using a non-destructive method based on laser spectroscopy (FT-System, Alseno, Italy). Each sample is placed in front of the laser source and set for the required centering option. The centering plate is spring-loaded to ensure correct bottle positioning. The laser beam crosses the headspace of the bottle at the point where the thickness is as homogeneous as possible to analyze the entire surface under examination thanks to the rotation of the bottle. The high-resolution analysis was performed to complete measurement in 20 s. At first, the system detects the temperature of the bottle and then carries out the measurement.

### 2.5. Colorimetric Analysis

A CR-400 portable colorimeter (Konica Minolta Sensing, Inc., Osaka, Japan) was used to measure the color parameters of apple juice at regular intervals up to 28 days (0, 7, 14, 21, and 28 days). The CIE colorimetric parameters were considered, including lightness (L*), redness-greenness (a*), and yellowness-blueness (b*). After the calibration of the instrument with the white standard (L = 99.36, a = −0.12, and b = −0.07), three readings were taken for each sample at room temperature and the measurement is expressed as the average of the three measurements. The total color variation (ΔE) was calculated for each sample according to Equation (1):

(1) $$\Delta E=\sqrt{{\left(\Delta L^{*}\right)}^{2}+{\left(\Delta a^{*}\right)}^{2}+{\left(\Delta b^{*}\right)}^{2}}$$

where ΔL*, Δa*, and Δb* are the differences between the corresponding color parameters of the samples during storage and those of samples at the beginning of the storage period (t0). Total color variation classification was used to analyze the data: 0.0–0.2 not perceptible, 0.2–0.5 very small, 0.5–1.5 small, 1.5–3.0 distinct, 3.0–6.0 very distinct, 6.0–12.0 great, >12 very great [29]. Yellowness Index (YI) was calculated according to the protocol Francis et al. [30] presented inEquation (2):

YI = 142.86 b*/L*.(2)

### 2.6. Statistical Analysis

The statistical analysis of data was carried out by one-way analysis of variance (one-way ANOVA) and the differences between means were analyzed with Duncan’s test (*p* < 0.05) using SPSS Statistics Ver. 20 software (IBM Corporation, New York, NY, USA).

## 3. Results and Discussion

### 3.1. Visual Appearance and Deformation Test of Bottles after HPP Treatment

**Figure 1:**
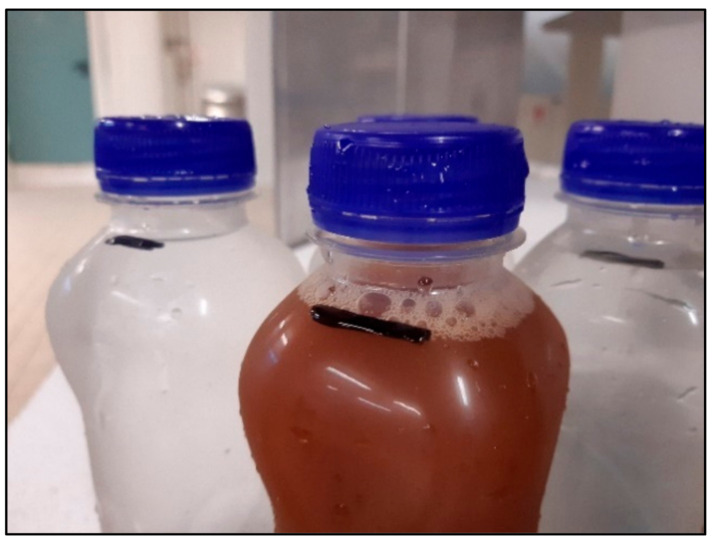
Visual appearance and hermeticity of PLA bottles filled with water and apple juice after subjecting to HPP treatment at 600 MPa for 3 min.

The suitability of a PLA bottle for HPP treatment should be primarily evaluated in terms of mechanical resistance, tightness of closure and cap/bottleneck mating, and ability to restore the original shape of the bottle after the pressurization treatment since HPP treatment submits packages to very high mechanical stress. Mechanical resistance and recovery of shape after pressurization is fundamental and must be assessed when evaluating a new packaging subjected to HPP, since high hydrostatic pressure can determine a volume reduction up to 19% [31]. In a preliminary test, a batch of 5 PLA bottles was filled with water and subjected to HPP treatment with no sign of loss of hermeticity or deformation. Similarly, the treatment of PLA bottles filled with apple juice at 600 MPa for 3 min did not cause alterations in the bottle shape and content, confirming the suitability of PLA bottles to withstand HPP conditions similar to PET bottles. The hermeticity and maintenance of the product level filled with water and apple juice after HPP treatment is presented in Figure 1. The initial content level was marked prior to HPP cycle and the same level was confirmed after the treatment. The verification of the product level assumed that, in the event of failure, water would enter the bottle from the pressurized chamber, altering the product level.

### 3.2. Microbiological Analyses

Microbiological analyses were carried out on the apple juice samples after HPP treatment at 600 MPa for 3 min to confirm the suitability of the applied conditions to reduce the microbial load, which arises from external contamination of the fruits, manipulation, and processing. Total mesophilic bacteria (TMB), yeasts, and molds (YMC) counts expressed as CFU/mL apple juice before and after HPP treatment are presented in Table 1. The initial load was moderate in the non-treated control sample and turned to not detectable after one HPP cycle. Results were in agreement with previous studies on fruit juice, which report the initial microbial load of about 10^4^ and post-treatment values below 10 CFU/mL [32]. Besides, microbiological stability was also assessed through monitoring TMB and YM counts during 28 days of storage (0, 7, 14, 21, and 28 days) at refrigerated conditions at 6 °C in samples packed in PET and PLA bottles (Table 1).

As it can be inferred, the YM count of the HPP-treated apple juice remained below the detection limit throughout the storage period for both PET and PLA bottles. TMB counts showed very low values, slightly above the limit of detection, hence they could be considered as negligible and not significantly different from the values (not detected) determined for PET. In any case, they did not show an increasing trend during storage, thus confirming the effective microbial stabilization by HPP treatment. The recovery of a few bacterial colonies may be ascribed to the germination of spores. Among the most resistant spore-forming bacteria, *Alicyclobacillus acidoterrestris* is often associated with spoilage of fruit juices [2,33]. Previous studies have reported that HPP is not effective at inactivating spore-forming bacteria, such as *Alicyclobacillus acidoterrestris,* especially when the pressurization is applied at low temperatures [2,34,35]. Similarly, fungal colonies were not detected in agreement with other studies, which proved that yeasts and molds are the most sensitive microorganisms to high hydrostatic pressures and can be inactivated even by modest HPP conditions [35,36]. According to U.S. Food and Drug Administration (FDA) regulation, 5-log reduction in the number of microbial cells in the fruit juices is required [37,38]. In summary, our results suggested that HPP treatment can be effectively applied as an alternative to pasteurization since it guarantees microbial stability in the medium-long term, thus allowing a refrigerated shelf life compatible with the needs of modern distribution.

### 3.3. Headspace Gas Composition

Headspace gas composition was analyzed through the conventional destructive approach and non-destructive method based on laser spectroscopy. The latter method has the advantage of monitoring the same samples without implying package piercing [39].

The conventional gas analysis was performed at regular intervals of up to 28 days by gas chromatography of headspace samples to evaluate the barrier performance of the PET and PLA bottles. After two hours from bottling in N_2_-saturated conditions, the headspace sampling revealed 2.8% O_2_. This level proves the incomplete substitution of air with N_2_ during bottling but can also be ascribed to the O_2_ equilibration between liquid and headspace. The juice is aerated and some O_2_ is solubilized in the liquid during juice pressing and pumping. The bottling in the N_2_-saturated atmosphere, then, minimizes the O_2_ level in the headspace and creates the conditions for a subsequent gas re-equilibration from the liquid to the headspace [40]. This phenomenon has been confirmed in the test carried out with the non-destructive approach, as it will be presented henceforth.

Vitamin C concentration in the juice during the storage can be predicted from the amount of oxygen presented in the headspace of the bottles [41]. Van Bree et al. [42] found a linear relationship between ascorbic acid degradation and the initial headspace oxygen concentrations in the fruit juice. The changes in sensory and nutritional properties of orange juice packed in PET bottles due to the oxidation of ascorbic acid were also reported by Bacigalupi et al. [41]. The headspace gas composition in both bottle types showed a substantial steady trend compared with the initial relative gas composition (Table 2).

Overall, the O_2_ headspace level was slightly reduced in the first 14 storage days, probably due to the O_2_ consumption by biochemical reactions. Considering the various phenomena involved in headspace gas variations (equilibrium liquid-gas, gas permeation, CO_2_ production, and O_2_ uptake due to microbial and biochemical reactions) [41], it can be concluded that the O_2_ level was not affected by the bottle type. It should be noted that the conventional gas measurement was characterized by certain data variability, probably due to the sampling operation (needle piercing of the plastic bottle).

Similarly, the CO_2_ levels after bottling and during storage were higher than atmospheric values. Such levels could not be attributed either to residual gas or permeation but were ascribed to CO_2_ equilibration in the headspace from dissolved CO_2,_ which was produced by fermentation in the time-lapse between pressing and HPP treatment. It must be highlighted that throughout processing and shipping, until bottling, the juice was kept at 2–4 °C. In these conditions, the gas solubilization in the liquid is favored and is maintained until a change in the equilibrium occurs.

**Figure 2:**
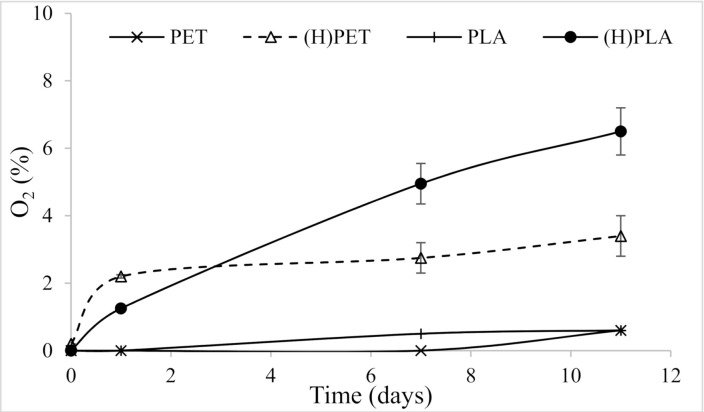
Time-course of O_2_ concentration as measured by laser spectroscopy in PLA and PET bottles, empty or filled with water (H).

The test carried out on bottles by the non-destructive laser spectroscopy-based method allowed to monitor the same sample bottles with no interference by possible air leakage during sampling. Our results showed that the liquid content of the bottle contributed to increasing the O_2_ headspace concentration, even if the initial gas level was set to zero. The data in Figure 2 showed that PLA and PET bottles filled with water had the highest O_2_ levels, while the empty bottles filled with N_2_ maintained very low O_2_ levels (<0.2%), irrespective of the polymer nature.

### 3.4. Color Parameters

The color of fruit juice is a key factor in quality perception by consumers, greatly affecting sensory acceptance [43]. The color parameters of fresh apple juice were 24.38, 3.43, and 4.81 for L*, a*, and b*, respectively. HPP treatment caused a decrease in L*, a*, and b* value (Table 3), indicating apple juice became slightly darker, less red, and less yellow. In the Hunter scale, L* measures the lightness and varies from 100 for perfect white to zero for black [44]. In this study, the L* value ranged from 22.30 to 26.43 in PET bottles and significantly increased during 28 days of storage. A similar trend was observed for the L* value of juice in PLA bottles, which significantly (*p* < 0.05) increased from 22.30 to 26.31. The increase in color lightness may be ascribed to a natural clarification of the juice during storage. This event is likely to take place in HP-treated juices since HPP is known to be ineffective or only partially effective on enzymes. Pectin methyl esterase (PME) catalyzes the de-esterification of pectins, which are abundant in apple juice and are responsible for their cloudiness. This enzyme is, therefore, recognized as responsible for the precipitation of pectins as calcium pectate [45]. PME is known to be very barotolerant [46], and its residual activity in HP-treated apple juice may, therefore, determine the reduction of cloudiness and sedimentation of suspended solids.

The positive a* values express the redness of color [44]. In this study, the a* value ranged from 2.56 to 2.65 in PET bottles and did not significantly (*p* > 0.05) change after 28 days of storage. A similar trend was observed for PLA bottles and this value ranged from 2.56 to 2.69, highlighting non-significant (*p* > 0.05) changes. The positive b* values express yellowness. Moreover, this color coordinate did not significantly change after 28 days of storage in PET bottles (3.90) while it was increased and reached 4.15 in PLA bottles (*p* < 0.05).

**Figure 3:**
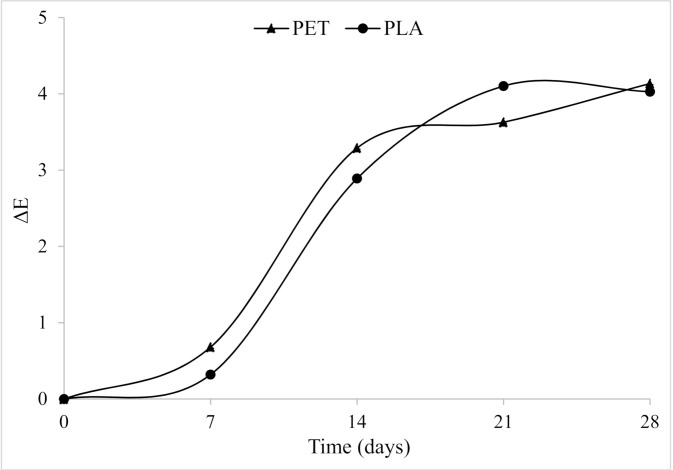
Overall color variation (ΔE) of apple juice during refrigerated storage at 6 °C in PLA and PET bottles.

Besides, ΔE was calculated for each packaging option (Figure 3). This parameter converts the color coordinates into an indicator that can be related to human perception. ΔE showed an exponential trend in the first 14 days, followed by a steady state until the end of observations, irrespective of the packaging type. Total color variation was very small up to seven days of refrigerated storage for both PET and PLA bottles ranging from 0.2–0.5, respectively. At the end of 28 days of storage, the total color difference for both PET and PLA bottles was similar and showed very distinct variations corresponding to 3.90.

It was observed that HPP is not effective at inactivating enzymatic activities, such as polyphenol oxidase [47], and the overall color variation observed during the storage period should be attributed to residual activities of polyphenol oxidase and PME, irrespective of the packaging conditions.

Yellowness index was also calculated (Table 3). This parameter is directly correlated with the quality of the product and consumer acceptance [48]. For example, carotenoids oxidation during storage leads to a decrease in yellowness [49]. In this study, the yellowness index of fresh apple juice was 28.18. HPP treatment did not significantly (*p* > 0.05) change this parameter for both PET and PLA bottles. Yellowness index generally slightly reduced in both PET and PLA bottles with some fluctuations during 28 days of storage (*p* < 0.05).

## 4. Conclusions

Today, consumer choices are driven both by quality-related factors and by environmental sustainability aspects, which are especially related to the packaging system. The reduction of environmental impacts arising from the packaging is an effective strategy for the overall sustainability improvement, especially for products characterized by a high packaging relative environmental impact, such as juices and beverages. In this context, premium fruit juices processed with non-thermal technologies are available, but the possibility to couple a green processing technology with a green packaging system has not been exploited yet. The present study assessed the feasibility of employing PLA bottles as an alternative to PET ones, for the packaging and subsequent HPP treatment of apple juice. In a perspective of improving food chain sustainability, PLA and other bioplastics may replace conventional plastics for some specific uses, such as fresh and minimally processed products, offering sufficient performances able to maintain the shelf life standards.

This study proved that PLA is a valid sustainable alternative to conventional PET bottles for the packaging and HPP treatment of apple juice due to its: (i) Biobased nature, (ii) compostability/recyclability, (iii) mechanical resistance and ability to restore the initial shape after HPP treatment, (iv) protection offered to the product, which is comparable with PET for short-term storage. The study also proved the effectiveness of HPP for the stabilization of juices and demonstrated the potential of non-destructive gas measurement systems for the verification of diffusional properties of bottles.

## Figures and Tables

**Table 1:** Total mesophilic bacteria (TMB) and yeast and molds (YM) in the control (untreated) and HPP-treated apple juice during 28 days of refrigerated storage.

	Time (days)	TMB (CFU/mL)	YM (CFU/mL)
Control (untreated)		1.18 ± 0.29 × 10^4^	6.00 ± 0.97 × 10^3^
	0	n.d.	n.d.
	7	4.55 ± 0.00	n.d.
PET	14	n.d.	1.50 ± 0.71
	21	n.d.	n.d.
	28	n.d.	n.d.
	0	n.d.	n.d.
	7	2.27 ± 3.21	n.d.
PLA	14	1.00 ± 1.41	n.d.
	21	1.00 ± 1.41	n.d.
	28	1.50 ± 0.71	n.d.

n.d.: not detected.

**Table 2:** Percent gas composition of the headspace of PET and PLA apple juice bottles. Values are presented as mean ± standard deviation.

	Time (days)	CO_2_ (%)	O_2_ (%)
PET	0	0.6 ± 0.8 ^B^	2.8 ± 0.4 ^A^
	7	2.0 ± 0.6 ^A^	3.0 ± 0.8 ^A^
	14	1.7 ± 0.0 ^AB^	1.7 ± 0.0 ^A^
	21	1.6 ± 0.4 ^AB^	2.8 ± 0.8 ^A^
	28	1.7 ± 0.0 ^AB^	2.0 ± 0.1 ^A^
PLA	0	0.6 ± 0.7 ^B^	2.8 ± 0.4 ^A^
	7	1.6 ± 0.3 ^AB^	1.4 ± 0.4 ^B^
	14	1.8 ± 0.2 ^A^	2.7 ± 0.6 ^A^
	21	1.5 ± 0.0 ^AB^	2.2 ± 0.4 ^AB^
	28	1.5 ± 0.2 ^AB^	2.8 ± 0.3 ^A^

Different capital letters show significant differences (*p* < 0.05) among storage times. No significant difference resulted among treatments for each storage time.

**Table 3:** Instrumental color parameters of HPP-treated apple juice during refrigerated storage at 6 °C in PLA and PET bottles. L*: lightness, a*: redness-greenness, b*: yellowness-blueness, YI: yellowness index. Values are presented as mean ± standard deviation.

	Time (days)	L*	a*	b*	YI
PET	0	22.30 ± 0.23 ^a E^	2.56 ± 0.04 ^a B^	3.90 ± 0.08 ^a B^	24.98 ± 0.91 ^a D^
	7	22.72 ± 0.26 ^a D^	2.83 ± 0.06 ^a A^	4.36 ± 0.12 ^a A^	27.42 ± 1.03 ^a E^
	14	25.29 ± 0.15 ^a C^	1.98 ± 0.07 ^b C^	2.66 ± 0.12 ^b A^	15.03 ± 0.75 ^a A^
	21	25.90 ± 0.21 ^b B^	2.61 ± 0.04 ^b B^	3.43 ± 0.07 ^b C^	18.92 ± 0.54 ^a B^
	28	26.43 ± 0.25 ^a A^	2.65 ± 0.05 ^a B^	3.90 ± 0.11^a B^	21.09 ± 0.80 ^a C^
PLA	0	22.30 ± 0.23 ^a E^	2.56 ± 0.04 ^a B^	3.90 ± 0.08 ^a B^	24.98 ± 0.91 ^a C^
	7	22.22 ± 0.39 ^a C^	2.71 ± 0.07 ^a A^	3.64 ± 0.11 ^b B^	23.40 ± 1.09 ^a BC^
	14	25.11 ± 0.19 ^a B^	2.23 ± 0.12 ^a C^	3.32 ± 0.13 ^a C^	18.89 ± 0.89 ^a A^
	21	26.40 ± 0.01 ^a A^	2.77 ± 0.06 ^a A^	3.73 ± 0.03 ^a B^	20.18 ± 0.15 ^a A^
	28	26.31 ± 0.53 ^a A^	2.69 ± 0.08 ^a AB^	4.15 ± 0.28 ^a A^	22.53 ± 1.96 ^a B^

Different capital letters show significant differences (*p* < 0.05) among storage times; different small letters show significant differences (*p* < 0.05) among treatments for each storage time.

## Data Availability

The data presented in this study are available on request from the corresponding author.
